# Defective Synapse Maturation and Enhanced Synaptic Plasticity in Shank2 Δex7^–/–^ Mice

**DOI:** 10.1523/ENEURO.0398-17.2018

**Published:** 2018-07-10

**Authors:** Stephanie Wegener, Arne Buschler, A. Vanessa Stempel, Sukjae J. Kang, Chae-Seok Lim, Bong-Kiun Kaang, Sarah A. Shoichet, Denise Manahan-Vaughan, Dietmar Schmitz

**Affiliations:** 1 Neuroscience Research Center, Charité Universitätsmedizin, 10117 Berlin, Germany; 2 Medical Faculty, Department of Neurophysiology, Ruhr University Bochum, 44801 Bochum, Germany; 3 School of Biological Sciences, Seoul National University, Seoul 08826, Korea; 4 Institute of Biochemistry and Cluster of Excellence NeuroCure, Charité Universitätsmedizin, 10117 Berlin, Germany; 5 Neuroscience Research Center, Cluster of Excellence NeuroCure, German Center for Neurodegenerative Diseases, Charité Universitätsmedizin, 10117 Berlin, Germany

**Keywords:** autism, LTP, maturation, PSD, shank, synapse

## Abstract

Autism spectrum disorders (ASDs) are neurodevelopmental disorders with a strong genetic etiology. Since mutations in human *SHANK* genes have been found in patients with autism, genetic mouse models are used for a mechanistic understanding of ASDs and the development of therapeutic strategies. SHANKs are scaffold proteins in the postsynaptic density of mammalian excitatory synapses with proposed functions in synaptogenesis, regulation of dendritic spine morphology, and instruction of structural synaptic plasticity. In contrast to all studies so far on the function of SHANK proteins, we have previously observed enhanced synaptic plasticity in Shank2 Δex7^−/−^ mice. In a series of experiments, we now reproduce these results, further explore the synaptic phenotype, and directly compare our model to the independently generated Shank2 Δex6-7^−/−^ mice. Minimal stimulation experiments reveal that Shank2 Δex7^−/−^ mice possess an excessive fraction of silent (i.e., α-amino-3-hydroxy-5-methyl-4-isoxazolepropionic acid, short, AMPA receptor lacking) synapses. The synaptic maturation deficit emerges during the third postnatal week and constitutes a plausible mechanistic explanation for the mutants’ increased capacity for long-term potentiation, both *in vivo* and *in vitro*. A direct comparison with Shank2 Δex6-7^−/−^ mice adds weight to the hypothesis that both mouse models show a different set of synaptic phenotypes, possibly due to differences in their genetic background. These findings add to the diversity of synaptic phenotypes in neurodevelopmental disorders and further support the supposed existence of “modifier genes” in the expression and inheritance of ASDs.

## Visual Abstract

**Figure:**
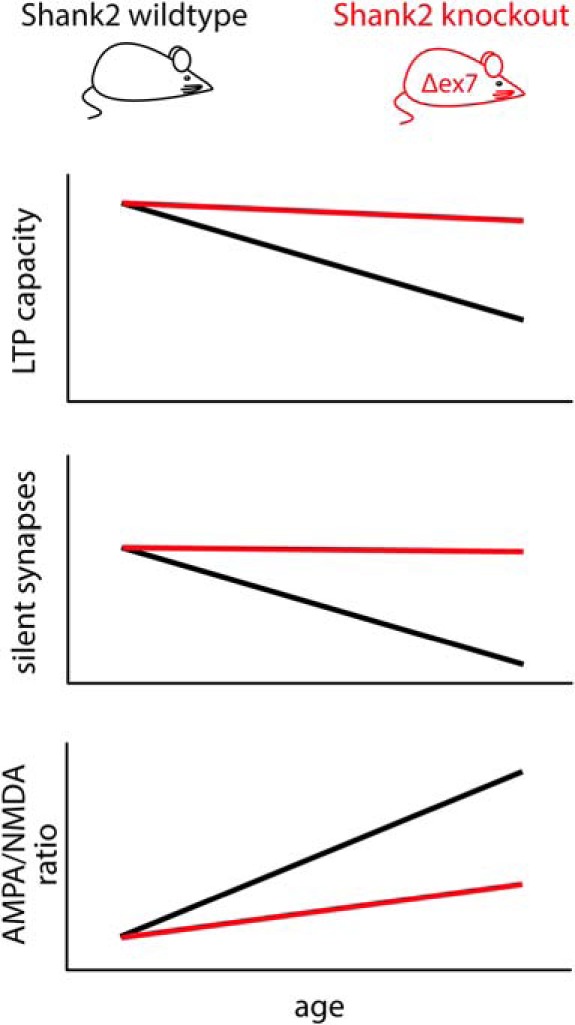

## Significance Statement

Autism spectrum disorders have a global prevalence of 0.1–2%, a fraction of which is caused by mutations in human *SHANK* genes. A number of Shank mouse models that reproduce behavioral symptoms also show reduced synaptic plasticity, which is why boosting plasticity has become one line of therapeutic rescue efforts. In contrast to all studies so far on the function of SHANK proteins, we observe enhanced plasticity in Shank2 Δex7^−/−^ mice and uncover a previously unrecognized synapse maturation deficit. This neurodevelopmental phenotype is shared among a number of mouse models for neurodevelopmental disorders, suggesting synapse maturation as a field of future studies and for the exploration of therapeutic intervention. The observation of distinct and noncongruent phenotypes in genetically similar yet nonidentical mouse models adds weight to the hypothesis that genetic interactions of putative “modifier genes” might influence the phenotype.

## Introduction

The activity-dependent formation and remodeling of synaptic connections is pivotal to adaptive neural circuit function. Dysregulation of these processes is considered a prime cause of neurodevelopmental diseases such as autism spectrum disorders (ASDs; Ebert and Greenberg, 2013). The list of mutations associated with ASDs and the number of mouse models is growing rapidly, yet understanding the connections among genetic mutation, synaptic defects, and disease phenotypes remains a challenge.

Mutations in all three *SHANK* genes (*SHANK1*, *SHANK2*, and *SHANK3*) occur in autistic patients (Durand et al., 2007; Berkel et al., 2010; Sato et al., 2012), and Shank mutant mice reproduce several autism-related phenotypes (Bozdagi et al., 2010; Peça et al., 2011; Wang et al., 2011; Schmeisser et al., 2012; Won et al., 2012; Lee et al., 2015; Peter et al., 2016). SHANKs (short for SH3 and multiple ankyrin repeat domains protein, also referred to as ProSAPs) are scaffold proteins in the postsynaptic density of mammalian excitatory synapses, linking postsynaptic membrane proteins to the cytoskeleton (for review, see Sheng and Kim, 2000) to serve functions in synaptogenesis (Du et al., 1998; Roussignol et al., 2005), regulation of dendritic spine morphology (Sala et al., 2001; Haeckel et al., 2008; Kim et al., 2009; Verpelli et al., 2011; Durand et al., 2012), and instruction of structural synaptic plasticity (MacGillavry et al., 2016).

Considerable efforts have been made to understand and differentiate the roles of different Shank isoforms in synaptic function and ASD pathophysiology (for review, see Jiang and Ehlers, 2013). The picture is complicated by diverging reports on the synaptic pathophysiology of mice lacking Shank2: for two independently generated Shank2 knock-out (KO) mice, noncongruent results on long-term plasticity and excitatory synaptic transmission were reported in two independent studies (Shank2 Δex7^−/−^, Schmeisser et al., 2012; Shank2 Δex6-7^−/−^, Won et al., 2012), and differences in inhibitory synaptic transmission were found in a direct comparison of the two mouse models (Lim et al., 2017).

To consolidate and mechanistically advance our understanding of excitatory synaptic transmission in Shank2 knock-out mice, we here report robustly increased long-term potentiation (LTP) in Shank2 Δex7^−/−^ mice upon electric stimulation *in vitro* and also *in vivo*. We find that Shank2 Δex7^−/−^ mice suffer from deficient synaptic maturation and an increased fraction of AMPA receptor-lacking synapses, suggesting a mechanistic explanation for their increased LTP capacity. A direct comparison of *in vivo* LTP in Shank2 Δex6-7^−/−^ and Shank2 Δex7^−/−^ mice reveals further differences between the two mouse models, supporting the idea of genetic interactions in the Shank2 mouse model (Lim et al., 2017), paralleling observations of putative modifier genes in the expression and inheritance of ASDs (Leblond et al., 2012).

## Materials and Methods

Shank2 Δex7 (Schmeisser et al., 2012) and Shank2 Δex6-7 mice (Won et al., 2012) were bred on a C57BL/6J background with a heterozygous breeding protocol. The study was conducted in accordance with the European Communities Council Directive of September 22, 2010 (2010/63/EU) for care of laboratory animals and after approval by the local ethics and/or animal welfare committees [Berlin animal experiment authorities and the Animal Welfare Committee of the Charité Berlin (File reference: T100/03), and Landesamt für Naturschutz, Verbraucherschutz und Umweltschutz, Nordrhein Westfalen, respectively]. Wild-type littermates were used as a control throughout, and experimenters were blind to the genotype of the tested animals for data collection and analysis.

**Figure 1.**
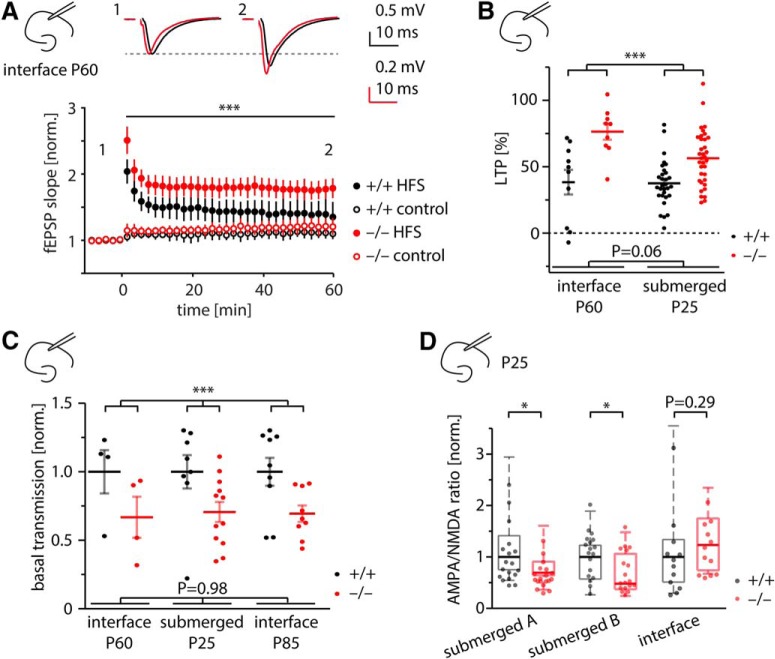
Enhanced LTP and reduced basal transmission in Shank2 Δex7^−/−^ mice *in vitro.* ***A***, Enhanced LTP in Shank2 Δex7^−/−^ mice *in vitro*. NMDA receptor-dependent LTP in the CA1 region of corticohippocampal slices was induced by high-frequency stimulation protocol (HFS; closed symbols). In a subset of experiments (+/+, 5/10; –/–, 7/9), synaptic responses of a nonpotentiated fiber tract were recorded as an additional control (control; open symbols). Example traces at top. ***p = 0.0025 [difference between genotypes in a two-way ANOVA; *n*(*N*)_+/+_ = 10(3), *n*(*N*)_−/−_ = 9(3)]. Slices were stored in an ACSF/oxygenized air interface chamber as reported by Won et al. (2012). ***B***, Increased LTP in Shank2 Δex7^−/−^ mice is irrespective of animal age and slice storage. Submerged data replotted from Schmeisser et al. (2012). Significance was tested with two-way ANOVA ***p < 0.0001 for genotype comparison across conditions. ***C***, Decreased basal synaptic transmission in Shank2 Δex7^−/−^ mice irrespective of animal age and slice storage. Basal transmission for each experiment is expressed as a single slope fitted to the input–output function of fEPSP slope vs fiber volley. Slopes of each group are normalized to the population mean of the wild type in the respective recording condition. Submerged data (P25) from the study by Schmeisser et al. (2012) are reanalyzed. Significance was tested with two-way ANOVA. ***p = 0.0005 for genotype comparison across conditions. ***D***, Slice storage conditions affect AMPA/NMDA receptor ratios. Significance was tested with Mann–Whitney *U* test. AMPA/NMDA receptor ratios are significantly reduced in CA1 pyramidal cells of Shank2 Δex7^−/−^ mice when slices are stored submerged in ACSF before recording [submerged 1: *p* = 0.013 data replotted from the study by Schmeisser et al., 2012; submerged 2: *p* = 0.036; see also Fig. 4*B*], but not when stored in an ACSF/oxygenated air interface chamber. Mouse age was 3-4 weeks for all groups.

Hippocampal brain slices were prepared from animals of both sexes as described previously (Schmeisser et al., 2012). Briefly, mice were anesthetized with isoflurane and decapitated. Brains were rapidly removed and transferred to ice-cold ACSF slicing solution. The ACSF slicing solution contained the following (in mm): 87 NaCl, 26 NaHCO_3_, 50 sucrose, 25 glucose, 3 MgCl_2_, 2.5 KCl, 1.25 NaH_2_PO_4_, and 0.5 CaCl_2_. The ACSF recording solution contained the following (in mm): 119 NaCl, 26 NaHCO_3_, 10 glucose, 2.5 KCl, 2.5 CaCl_2_, 1.3 MgCl_2_, and 1 NaH_2_PO_4_. All ACSF was equilibrated with carbogen (95% O_2_, 5% CO_2_). Tissue blocks containing the hippocampus were mounted on a Vibratome (VT1200, Leica) and cut into horizontal slices of 300 μm. For submerged slice storage (used for minimal stimulation experiments), slices were, after preparation, kept submerged in ACSF at 34°C for 30 min, then slowly cooled to room temperature where they were left to recover for at least 30 min up to 5 h. Recordings were performed in slices submerged in ACSF and at room temperature. For a subset of experiments (Fig. 1), we reproduced a range of conditions from the study by Won et al. (2012): immediately after preparation, slices were transferred into an ACSF/oxygenated air interface chamber and allowed to remain there to recover until recording, for at least 1 h and at most 5 h. Recordings were performed in a submerged recording chamber; the storage and recording temperature for these experiments was 34°C. Mouse age for *in vitro* experiments was 8-9 weeks for the experiments shown in Figure 1*A* and 3–4 weeks for experiments in Figure 1*D* and Figure 4*A–D*. For the remaining *in vitro* experiments (Figs. 1*B*,*C*, 4*E*), mouse age is indicated in the figure legend.

For all *in vitro* experiments, data were recorded with an Axopatch 700A Amplifier (Molecular Devices), digitized at 5 kHz, filtered at 2 kHz, and recorded in IGOR Pro 4.0. Evoked postsynaptic responses were induced by stimulating Schaffer collaterals in CA1 stratum radiatum. Field EPSPs (fEPSPs) were recorded in stratum radiatum. fEPSP rising slopes were fitted to 20–80% of the fEPSP amplitude. LTP was induced by a single tetanus of 100 pulses at 100 Hz. For whole-cell patch-clamp recordings, the recording ACSF was supplemented with 1 μm gabazine. Pipettes had resistances of 2–3 MΩ. Liquid junction potential was not corrected. Series resistance (not compensated) was constantly monitored and was not allowed to increase beyond 22 MΩ or change by >20% during the experiment. Compound EPSCs were recorded at −60 and +40 mV with a cesium-based intracellular recording solution containing the following (in mm): 145 CsCl, 10 HEPES, 0.2 EGTA, 2 MgCl_2_, 2 NaATP, 0.5 NaGTP, and 5 phosphocreatine, with osmolarity of 305 mOsm and pH adjusted to 7.2 with CsOH. The AMPA receptor-mediated component of the EPSC was estimated by measuring the peak amplitude of the averaged EPSC at −60 mV. The n-methyl-d-aspartate, short, NMDA receptor-mediated component was estimated at +40 mV by measuring the amplitude of the averaged EPSC 25 ms after stimulation.

For minimal stimulation, the stimulation frequency was 0.2 Hz, and the stimulation electrode was placed to produce a single-peak response. At +40 mV holding potential, stimulation intensity was reduced until transmission failures were observed (in ∼10–40% of events), and 20–50 events were recorded. Cells were subsequently clamped to −60 mV holding potential, and 30–50 events were recorded at the same stimulation intensity. In a subset of experiments, this order was reversed [i.e., stimulation intensity was adjusted and miniature EPSCs (minEPSCs) were recorded at −60 mV first, before cells were clamped to +40 mV]. We did not observe systematic differences in failure rates (*r_f_*) or amplitudes of minEPSCs between the two regimes. Experiments with linearly increasing or decreasing failure rates and/or minEPSCs amplitudes at any holding potential were excluded from the analysis. *Post hoc* analysis counted a failure at depolarized potentials whenever the minEPSCs charge 5–40 ms after stimulation did not exceed a threshold of 0.9 pC. Failures at hyperpolarized potentials were defined as events with an minEPSCs peak smaller than twice the signal noise (i.e., the SD of the signal in a 3 ms time window averaged over all sweeps at a certain holding potential) of that recording. The average signal noise was not different among experimental groups, and experiments with high background noise were excluded from the analysis. While the absolute failure rates depended on how the criteria for failure versus successes were set, the relative difference between genotypes did not. For each experiment, the synaptic potency was defined as the average amplitude of all minEPSCs at a given holding potential that qualified as successes. For EPSCs recorded at −60 mV, the amplitude was taken at the peak of all individual EPSCs after subtraction of the average failure (for removal of the stimulus artifact). For EPSCs recorded at +40 mV, the amplitude was read 25 ms after stimulation. Synaptic transmission under minimal stimulation can be described by a Poisson distribution: $P(k)\;\text{=}\;(m^{k}e^{-m})\text{/}k!$ with P(k) being the probability of k quanta being released and m being the mean quantal content. Since the failure rate $r_{f}\;\text{=}\;P(\text{0})\;\text{=}\;e^{-m}$, the fraction of silent synapses can be estimated as $\text{1}\;-\;\text{ln}(r_{f\;\text{−60mV}}\;-\;r_{f\;\text{+40mV}})$ (Liao et al., 1995) and the synaptic potency $S\;\text{=}\;\text{−ln}\;(r_{f})\;\text{*}\;q$, with q being the mean quantal size.

**Figure 2.**
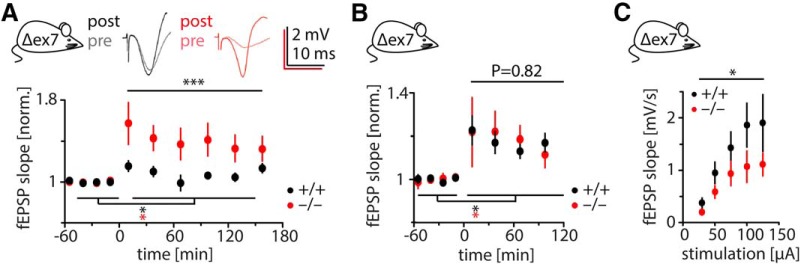
Enhanced LTP and reduced basal transmission in Shank2 Δex7^−/−^ mice *in vivo*. ***A***, Enhanced LTP in Shank2 Δex7^−/−^ mice *in vivo*. 3h-LTP was induced at time point “0” by high-frequency stimulation in awake, freely behaving mice (for details, see Materials and Methods). Example traces are at top. LTP was induced in both genotypes (ANOVA; *: +/+: *p* = 0.017, *N* = 16; −/−: *p* < 0.0001, *N* = 15) but was significantly larger in Shank2 Δex7^−/−^ mice (***p < 0.0001, difference between genotypes in a two-way ANOVA). ***B***, 2h-LTP was induced by mild high-frequency stimulation in awake, behaving mice (for details, see Materials and Methods). Significance was tested with two-way ANOVA. LTP was induced in both genotypes (*: +/+, *p* < 0.0001, *N* = 16; −/−, *p* = 0.0019, *N* = 16) with no significant difference between genotypes. ***C***, Basal synaptic transmission in awake, behaving mice is reduced in Shank2 Δex7^−/−^ mice compared with wild-type controls. *p = 0.011 (difference between genotypes in a two-way ANOVA; *N*_+/+_ = 12; *N*_−/−_ = 8).

**Figure 3.**
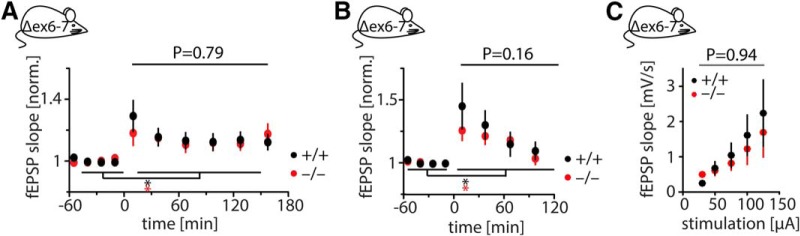
*In vivo* LTP in Shank2 Δex6-7^−/−^ mice. ***A***, High-frequency stimulation at time point “0” successfully induced 3h-LTP in Shank2 Δex6-7^−/−^ and wild-type mice (*: +/+, *p* < 0.0001, *N* = 10; −/−, *p* = 0.012, *N* = 10) with no detectable difference between genotypes. Significance was tested by two-way ANOVA. ***B***, 2h-LTP was successfully induced in both genotypes (*; +/+, *p* < 0.0001, *N* = 10; −/−, *p* < 0.001, *N* = 10). A trend for reduced potentiation in Shank2 Δex6-7^−/−^ mice did not reach significance (*p* = 0.16, difference between genotypes in a two-way ANOVA). ***C***, Basal synaptic transmission in awake, behaving mice is not significantly different in Shank2 Δex6-7^−/−^ mice compared with wild-type controls (*p* = 0.94, difference between genotypes in a two-way ANOVA; *N*_+/+_ = *N*_−/−_ = 5).

For *in vivo* experiments, 7- to 8-week-old male mice were implanted (under anesthesia) with a stimulation electrode in the Schaffer collaterals (2.0 mm posterior and 2.0 mm lateral to bregma) and a recording electrode in the stratum radiatum of the dorsal CA1 region (1.9 mm posterior and 1.4 mm lateral to bregma), as described previously (Buschler et al., 2012). Animals recovered for ∼10 d before experiments were commenced. Before each experiment, input/output (I/O) properties were recorded by increasing the stimulation intensity stepwise (Fig. 2*C*). Experiments were conducted in recording chambers [20 (length) × 20 (width) × 30 (height) cm] where animals could move freely and had access to food and water *ad libitum*. During recordings, implanted electrodes were connected via a flexible cable and a rotatable commutator to the stimulation unit and amplifier. Test-pulse stimulation was set to elicit 40% of the maximal I/O response. Stimuli of 0.2 ms duration were applied at a frequency of 0.025 Hz and recorded with a sample rate of 10 kHz. Different protocols were used to elicit LTPs of differing magnitudes and durations (Buschler et al., 2012), all NMDA receptor dependent (Ballesteros et al., 2016). Two induction protocols were found to elicit robust LTP that was stable for >3 h (3h-LTP). Protocol 1 contained four trains of 50 pulses at 100 Hz with a 5 min intertrain interval (Shank2 Δex7: *N*_+/+_ = *N*_−/−_ = 8; Shank2 Δex6-7: *N*_+/+_ = *N*_−/−_ = 5). Protocol 2 contained 2 trains of 50 pulses at 200 Hz with 5 min intertrain interval (Shank2 Δex7: *N*_+/+_ = 8, *N*_−/−_ = 7; Shank2 Δex6-7: *N*_+/+_ = *N*_−/−_ = 5). Results from both protocols were quantitatively similar and pooled for Figures 2*A* and 3*A* , respectively. Two protocols elicited LTP that was short lived, receding back to baseline in ∼2 h (2h-LTP): protocol 3, a single train of 50 pulses at 100 Hz stimulation (Shank2 Δex7: *N*_+/+_ = *N*_−/−_ = 8; Shank2 Δex6-7: *N*_+/+_ = *N*_−/−_ = 5); and protocol 4, two trains of 50 pulses at 100 Hz stimulation with a 5 min intertrain interval (Shank2 Δex7: *N*_+/+_ = *N*_−/−_ = 8; Shank2 Δex6-7: *N*_+/+_ = *N*_−/−_ = 5). Results from protocols 3 and 4 were quantitatively similar and pooled for Figures 2*B* and 3*B*, respectively. Statistical tests for the expression of LTP within an experimental group involved comparing post-tetanus responses to pretetanus baseline responses with time as a continuous variable (with all time points before induction set to 0 min) and preinduction/postinduction as a categorical variable. Tests between genotypes compared postinduction time points only, with time as a continuous variable and genotype as a categorical variable. Potentiation in text and Table 1 was quantified at 2–3 h postinduction for 3h-LTP and 1–2 h postinduction for 2h-LTP.

**Table 1.** Details of statistical analyses presented in the article

Figures	Experiment	*n*(*N*)	Test used	*p* value	Mean (CI)^*^
1A	*In vitro*, interface, P55	WT: 10(3), KO: 9(3)	ANOVA	0.0025	see 1B_left_
1B	Left: *in vitro*, interface, P55	WT: 10(3), KO: 9(3)	Two-way ANOVA	Experimental condition: 0.89 genotype: <0.0001	WT: 38.3 (18.2), KO: 76.4 (12.0)
	Right: *in vitro*, submerged, P25	WT: 30(5), KO: 34(6)			WT: 37.6 (6.1), KO: 56.3 (6.9)
1C	Left: *in vitro*, interface, P25	WT: 4(2), KO: 4(2)	Two-way ANOVA	Experimental condition: 0.98 genotype: 0.0005	WT: 1.00 (0.31), KO: 0.67 (0.29)
	Middle: *in vitro*, submerged, P25	WT: 8(3), KO: 11(4)			WT: 1.00 (0.24), KO: 0.71 (0.14)
	Right: *in vitro*, interface, P85	WT: 9(3), KO: 9(3)			WT: 1.00 (0.20), KO: 0.69 (0.12)
1D*	Left: *in vitro*, submerged, P25	WT: 18(8), KO: 19(6)	Mann–Whitney *U*	0.013	WT: 1.0 [0.8 1.4], KO: 0.7 [0.6 0.9]
	Middle: *in vitro*, submerged, P25	WT: 20(6), KO: 18(8)	Mann–Whitney *U*	0.036	WT: 1.0 [0.6 1.2], KO: 0.5 [0.4 1.1]
	Right: *in vitro*, interface, P25	WT: 12(2), KO: 12(2)	Mann–Whitney *U*	0.29	WT: 1.0 [0.5 1.3], KO: 1.2 [0.7 1.7]
2A	*In vivo*, P50, 3h-LTP	WT: 16, KO: 15	For LTP induction: two-way ANOVA	LTP (categorical): WT: 0.017, KO: <0.0001 time (continuous): WT: 0.42, KO: 0.005	LTP 2-3h postinduction (%): WT: 9.1 (8.6), KO: 32.6 (25.5)
			Between genotypes: two-way ANOVA	genotype: <0.0001 time: 0.016	
2B	*In vivo*, P50, 2h-LTP	WT: 16, KO: 16	For LTP induction: two-way ANOVA	LTP (categorical): WT: <0.0001, KO: 0.002 time (continuous): WT: 0.02, KO: 0.15	LTP 1-2h postinduction (%): WT: 15.0 (6.9), KO: 15.0 (12.1)
			Between genotypes: two-way ANOVA	Genotype: 0.82 time: 0.048	
2C	*In vivo*, P50	WT: 12, KO: 8	Two-way ANOVA	Genotype: 0.011 stimulation: <0.0001	WT_125μA_: 1.9 (1.1), KO_125μA_: 1.1 (0.5)
3A	*In vivo*, P50, L-LTP	WT: 10, KO: 10	For LTP induction: two-way ANOVA	LTP (categorical): WT: <0.0001, KO: 0.012 time (continuous): WT: 0.007, KO: 0.74	LTP 2-3h postinduction (%): WT: 12.9 (11.2), KO: 14.3 (12.0)
			Between genotypes: two-way ANOVA	Genotype: 0.79 time: 0.17	
3B	*In vivo*, P50, E-LTP	WT: 10, KO: 10	For LTP induction: two-way ANOVA	LTP (categorical): WT: 0.0002, KO: <0.0001 time (continuous): WT: 0.022, KO: 0.003	LTP 1-2h postinduction (%): WT: 12.0 (18.6), KO: 10.6 (13.2)
			Between genotypes: two-way ANOVA	Genotype: 0.16 time: 0.006	
3C	*In vivo*, P50	WT: 5, KO: 5	Two-way ANOVA	Genotype: 0.94 stimulation: <0.0001	WT_125μA_: 2.2 (1.9), KO_125μA_: 1.7 (1.4)
4A	*In vitro*, submerged, P25	WT: 11(6)	Student’s *t* test (paired)	WT: 0.19	WT_−60mV_: 16.3 (6.6), WT_+40mV_: 13.2 (5.6)
		KO: 14(6)	Student’s *t* test (paired)	KO: 0.0003	KO_−60mV_: 36.3 (10.9), KO_+40mV_: 12.7 (4.1)
4C	*In vitro*, submerged, P25	WT: 11(6), KO: 13(6)	Student’s *t* test	0.0018	WT: 3.1 (4.4), KO: 23.6 (9.4)
4D*	*In vitro*, submerged, P25	WT: 20(6), KO: 18(8)	Mann–Whitney *U*	0.036	WT: 1.9 [1.1 2.3], KO: 0.9 [0.7 2.0]
4E	*In vitro*, submerged, AMPA/NMDA ratio^*^	P13–P14: WT: 16(4) KO: 18(5) P21–P24: WT: 10(3), KO: 11(3) P25–P28: WT: 10(4), KO: 7(3)	Over age groups: Kruskal–Wallis test	WT: 0.0026, KO: 0.056	P13–P14: WT: 0.68 [0.47 0.88], KO: 0.45 [0.31 0.64] P21–P24: WT: 0.75 [0.55 1.09], KO: 0.63 [0.43 0.93] P25–P28: WT: 1.20 [0.88 1.48], KO: 0.78 [0.50 1.00]
			Between genotypes: Mann–Whitney *U*	P13–P14: 0.055 P21–P24: 0.083 P25–P28: 0.016	
	*In vitro*, submerged, LTP	P13–P14: WT: 10(2), KO: 7(2) P21–P24: WT: 15(2), KO: 14(2) P25–P28: WT: 15(3), KO: 20(3)	Over age groups: ANOVA	WT: 0.012, KO: 0.77	P13–P14: WT: 51.6 (12.9), KO: 61.6 (11.4) P21–P24: WT: 44.6 (7.9), KO: 58.2 (13.2) P25–P28: WT: 30.5 (8.2), KO: 55.3 (7.8)
			Between genotypes: Student’s *t* test	P13–P14: 0.30 P21–P24: 0.09 P25–P28: 0.0002	

* Median and percentiles [25th 75th] are reported for nonparametric datasets instead of mean and CI values.

Analyses were performed using custom-written procedures in IGOR Pro and MATLAB. Data in graphs and text are, unless stated otherwise, presented as the mean ± SE for parametric data and the median [25th 75th percentile] for nonparametric data (graphically, whiskers additionally represent the minimum and maximum values). Unpaired two-tailed Student’s *t* test (short: Student’s *t* test) and ANOVAs were used to test for the statistical significance of parametric data, and Mann–Whitney *U* tests and Kruskal–Wallis tests were used for nonparametric data. Results were considered to be significant at *p* < 0.05. Curve fitting was performed in MATLAB using a nonlinear least-squares algorithm. Stimulus artifacts were blanked or cropped in sample traces. Sample sizes are given as the number of experiments and the number of animals (*N*).

## Results

Two parallel studies on genetically similar Shank2^−/−^ mice have reported increased LTP (Schmeisser et al., 2012) and decreased LTP (Won et al., 2012), respectively, in hippocampal brain slices of Shank2-null mutants. To investigate whether biological or methodological differences account for this discrepancy, we first investigated in Shank2 Δex7^−/−^ mice (Schmeisser et al., 2012) how differences in slice storage, recording temperature, and animal age might affect the expression of LTP. To this end, we reproduced a range of conditions from the study by Won et al. [2012; i.e., slices were stored in an ACSF/oxygenated air (Haas type) interface chamber instead of submerged in ACSF before recording; animal age was 8–9 weeks instead of 3–4 weeks, and recordings were performed at elevated temperature instead of room temperature]. Hippocampal slices from Shank2 Δex7^−/−^ mice were then subjected to the common LTP induction protocol (for details, see Materials and Methods). In our hands, also in these conditions, Shank2 Δex7^−/−^ mice showed markedly higher LTP than wild-type controls [WT, 38 ± 9%; KO, 76 ± 6%; *N*_+/+_ = 10(3); *N*_−/−_ = 9(3); Fig. 1*A*].

Since the studies on Shank2 Δex7^−/−^ and Shank2 Δex6-7^−/−^ mice also reported different phenotypes with respect to excitatory basal synaptic transmission and synaptic AMPA/NMDA ratios, we assessed these physiologic parameters as well. A quantitative comparison between results from the previous study (Schmeisser et al., 2012) and new experiments is presented in Figure 1*B–D*. Slice storage affected phenotypes to varying degrees: While in our hands storing slices submerged in ACSF versus a Haas-type interface chamber before recording had no effect on the expression of LTP (*p* = 0.89 for comparison of experimental conditions in a two-way ANOVA; Fig. 1*B*) or basal synaptic transmission (*p* = 0.98 for comparison of experimental conditions in a two-way ANOVA; Fig. 1*C*), it did affect AMPA/NMDA receptor ratios (Fig. 1*D*), with a trend toward an increased AMPA/NMDA ratio in slices of Shank2 Δex7^−/−^ mice that have been stored in an interface chamber (Mann–Whitney *U* test, *p* = 0.29) compared with significantly reduced AMPA/NMDA ratio in slices that have been stored in submerged conditions (Mann–Whitney *U* test, *p* = 0.013 and *p* = 0.036 for two independent datasets; Fig. 1*D* ). Increased AMPA/NMDA ratios have been reported in Shank2 Δex6-7^−/−^ mice (Won et al., 2012); we thus conclude that the experimental conditions examined here might explain some diverging results in Shank2 Δex7^−/−^ versus Shank2 Δex6-7^−/−^ mice, like AMPA/NMDA ratios, but not the contrary observations on basal synaptic transmission and LTP.

In light of these results and reports on substantial changes in synaptic spine morphology after brain slice preparation (Kirov et al., 1999), we reasoned that an *in vitro* examination of Shank2^−/−^ phenotypes might be problematic, given that SHANK2 has a well described role in synaptogenesis and the regulation of structural dynamics in dendritic spines (MacGillavry et al., 2016). More precisely, we still wondered whether the decreased basal synaptic transmission and increased LTP we consistently observed in Shank2 Δex7^−/−^ mice might be secondary to slicing-induced synaptic remodeling. This motivated us to examine synaptic transmission and NMDA receptor-dependent LTP *in vivo*. Using established experimental procedures (Buschler et al., 2012; for details, see Materials and Methods), we compared Shank2 Δex7^−/−^ and wild-type mice with regard to their capacity to express LTP *in vivo*. We tested different induction protocols, eliciting both short- and long-lasting forms of LTP in freely behaving mice (Buschler et al., 2012), from here on referred to as 2h-LTP and 3h-LTP, respectively (for details, see Materials and Methods). Both forms of LTP could be elicited in Shank2 Δex7^−/−^ mice and wild-type controls (Fig. 2*A*,*B*), validating that synapses without SHANK2 can express LTP, as our data from acute slices suggest. For 3h-LTP, the potentiation in Shank2 Δex7^−/−^ mice significantly exceeded that of wild-type controls [WT, 9 ± 4% (*N* = 15); KO, 33 ± 13% (*N* = 16); *p* < 0.0001 in a two-way ANOVA; Fig. 2*A*]. Further corroborating our *in vitro* results, basal synaptic transmission was significantly decreased in Shank2 Δex7^−/−^ mice versus wild-type controls when assessed in awake, behaving animals (Fig. 2*C*). In summary, we observe enhanced LTP and reduced synaptic basal transmission in Shank2 Δex7^−/−^ mice under a range of different conditions both *in vitro* and *in vivo*.

In contrast to Shank2 Δex7^−/−^ mice, Shank2 Δex6-7^−/−^ mice have been reported to show decreased LTP *in vitro* (Won et al., 2012; Peter et al., 2016), and genetic differences between the two mouse models have been suggested to account for this discrepancy (Lim et al., 2017). However, a direct comparison of excitatory synaptic transmission in the two models has so far been lacking. Thus, we next investigated *in vivo* LTP in freely behaving Shank2 Δex6-7^−/−^ mice. Both short- and long-lasting forms of LTP could be induced in wild-type as well as knock-out mice, with no significant difference in the magnitude of LTP expression (Fig. 3). A trend toward reduced short-lasting LTP in Shank2 Δex6-7^−/−^ mice was not stable over time (2h-LTP: WT, 12 ± 10% (*N* = 10); KO, 11 ± 7% (*N* = 10); *p* = 0.16 in a two-way ANOVA; Fig. 3*B*), although it was reminiscent of *in vitro* observations made by other laboratories (Won et al., 2012; Peter et al., 2016).

**Figure 4.**
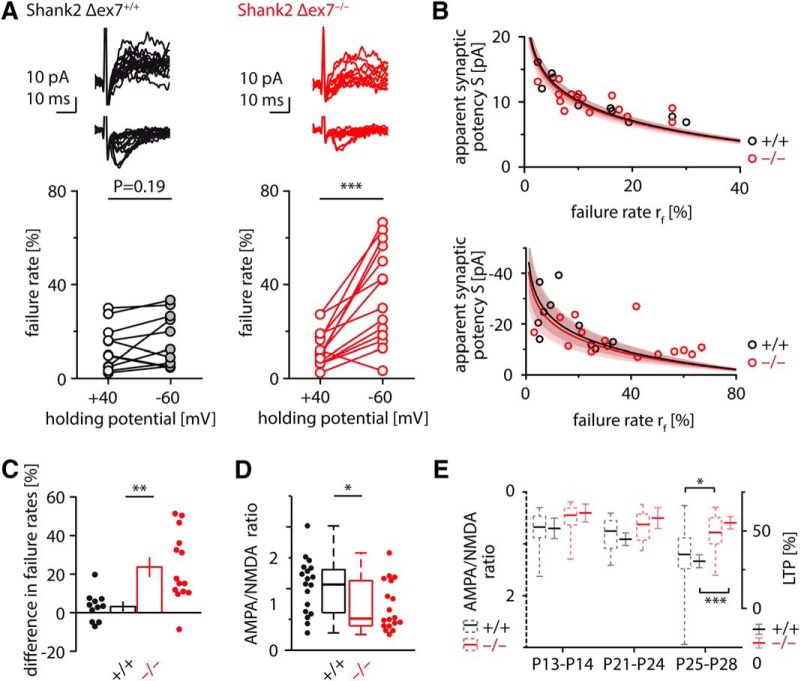
Minimal stimulation reveals insufficiently matured synapses in Shank2 Δex7^−/−^ mice. ***A***, EPSCs were recorded at different holding potentials under minimal stimulation *in vitro*. Failure rates are plotted for control (left) and Shank2 Δex7^−/−^ mice (right). ***p = 0.0003 [paired Student’s *t* test. Example traces at top (top, EPSCs recorded at +40 mV; bottom, EPSCs recorded at −60 mV). ***B***, For each holding potential, the apparent synaptic potency S (the average amplitude of all EPSCs) is expressed as a function of the failure rate *r_f_*. Circles, line, and shaded area in black and red represent individual experiments, best fit, and the 95% confidence interval for wild-type and Shank2 Δex7^−/−^ mice, respectively (for details, see Materials and Methods). ***C***, The respective difference between failure rates at hyperpolarized vs depolarized potentials (*r_f_*_−60mV_ − *r_f_*_+40mV_) is significantly higher in Shank2 Δex7^−/−^ mice than in wild-type controls. **p = 0.0018 (Student’s *t* test. ***D***, AMPA/NMDA receptor ratios calculated from the average EPSC of minimal stimulation experiments are smaller in Shank2 Δex7^−/−^ mice than in wild-type controls (average is calculated across successes and failures alike). *p = 0.036 Mann–Whitney *U* test. ***E***, Synaptic maturation in wild-type (black) and Shank2 Δex7^−/−^ mice (red) assessed in juvenile (P13–P14) and adolescent mice (P21–P28). Box plots (dashed) show AMPA/NMDA ratios from minimal stimulation experiments (left axis); mean and SE (nondashed) show LTP magnitude after tetanic stimulation (right axis). Significant differences can first be detected in mice aged P25–P28 (AMPA/NMDA ratios, *p* = 0.016, Mann–Whitney *U* test; LTP, *p* = 0.0002, Student’s *t* test).

How can we understand the phenomena of reduced synaptic transmission and increased LTP in Shank2 Δex7^−/−^ mice? Is there a mechanistic explanation linking these two findings? In Shank3^−/−^ mice, reduced LTP has been associated with NMDA as well as AMPA receptor hypofunction (Wang et al., 2011; Kouser et al., 2013). Both mechanisms seem possible, since hippocampal CA1 LTP is dependent on both NMDA and AMPA receptors in its induction and expression, respectively (Nicoll, 2017). Shank2 Δex7^−/−^ mice show reduced AMPA receptor-dependent basal synaptic transmission (Fig. 1*C*), and, in submerged stored slices, a reduction in AMPA/NMDA ratios (Fig. 1*D*). In *Drosophila melanogaster*, animals lacking all SHANK isoforms show synaptic maturation deficits at the glutamatergic neuromuscular junction (Harris et al. 2016). We thus set out to test whether synapses lacking SHANK2 might possess fewer AMPA receptors or be functionally silent by lacking them altogether, rendering these synapses salient LTP substrates. To estimate the fraction of silent synapses, we performed minimal stimulation experiments. For each set of stimulated synapses, we recorded EPSCs at a holding potential of −60 mV (conducted by AMPA receptor-containing synapses) and at +40 mV, when synapses lacking AMPA receptors but harboring NMDA receptors can also pass currents. Indeed, minimal stimulation revealed markedly higher failure rates (*r_f_*) in Shank2 Δex7^−/−^ mice than in wild-type controls at −60 mV but not at +40 mV [*r_f_*_−60mV_: WT, 16 ± 3%; KO, 36 ± 6%; *r_f_*_+40mV_: WT, 13 ± 3%; KO, 13 ± 2%; *N*_+/+_ = 11(6), *N*_−/−_ = 14(6); Fig. 4*A*], corresponding to a fraction of ∼51% silent synapses in knock-out mice, compared with ∼11% in wild-type controls. We next compared the transmission strength of individual synapses between Shank2 Δex7^−/−^ mice and wild-type mice. To that end, we expressed the apparent synaptic potency *S* (the average amplitude of successfully evoked EPSCs) as a function of the observed failure rate *r_f_* (which relates to the number of potentially active synapses) and fitted the mean quantal size q with $p\;\text{=}\;\text{−ln}\;(r_{f})\;\text{*}\;q$ (for details, see Materials and Methods). The average synaptic response estimated from this relationship was not different between Shank2 Δex7^−/−^ and wild-type mice for either NMDA receptor-mediated or AMPA receptor-mediated events (mean ± CI; *q*_NMDA_: WT, 4.4 ± 0.4 pA; KO, 4.3 ± 0.5 pA; *q*_AMPA_: WT, −9.7 ± 2.7 pA; KO, −8.8 ± 3.1 pA; Fig. 4*B*). This suggests that it is mainly the higher difference in failure rates in Shank2 Δex7^−/−^ mice (Fig. 4*C*), and thus an excess of silent synapses, that accounts for the reduced AMPA/NMDA receptor ratio of the knockout (Fig. 4*D*).

It is conceivable that the increased fraction of silent synapses in Shank2 Δex7^−/−^ mice provides the structural framework for the increased LTP. To investigate how the two phenomena are correlated through maturation of the hippocampal circuitry, we repeated minimal stimulation and LTP experiments in juvenile mice [postnatal day 13 (P13) to P14]. At that age, wild-type and Shank2 Δex7^−/−^ mice alike showed high failure rates at hyperpolarized holding potentials and, consequently, a high fraction of silent synapses [silent synapses: WT, ∼48%; KO, ∼52%; *r_f_* at −60 mV: WT, 43 ± 6%; KO, 42 ± 6%; *r_f_* at +40 mV: WT, 19 ± 2%; KO, 17 ± 2%; *N*_+/+_ = 16(7), *N*_−/−_ = 18(7); *t* test over difference in failure rates between genotypes, *p* = 0.76]. Within the same age range, the magnitude of LTP evoked through tetanic stimulation was comparably high in both genotypes [WT, 52 ± 7%; KO, 62 ± 6%; *N*_+/+_ = 10(2); *N*_−/−_ = 7(2); *p* = 0.3, Student’s *t* test], and AMPA/NMDA ratios were comparably low [WT, 0.71 ± 0.08; KO, 0.55 ± 0.08; *N*_+/+_ = 16(7); *N*_−/−_ = 18(7); *p* = 0.055, Mann–Whitney *U* test]. From P13 to P28, we saw significant synaptic maturation in juvenile wild-type mice (LTP, *p* = 0.01, ANOVA; AMPA/NMDA ratios, *p* = 0.003, Kruskal-Wallis) that was virtually absent in Shank2 Δex7^−/−^ mice (LTP, *p* = 0.77, ANOVA; AMPA/NMDA ratios, *p* = 0.06, Kruskal–Wallis test; Fig. 4*E*). We thus propose the deficient maturation of excitatory synapses in Shank2 Δex7^−/−^ mice as a possible cause for their decreased synaptic basal transmission, their decreased AMPA/NMDA ratios, and their increased capacity for LTP.

## Discussion

In summary, we consistently observe increased LTP in hippocampal Schaffer collateral–CA1 synapses of Shank2 Δex7^−/−^ mice *in vitro* as well as in awake behaving animals. We have further uncovered a developmental synapse phenotype, an excess of silent synapses, that could link the phenomena of decreased synaptic transmission and increased LTP. During LTP expression, synaptic strength increases with the incorporation of AMPA receptors, a process through which immature, silent synapses (that previously lacked such receptors) can become unsilenced (Isaac et al., 1995; Liao et al., 1995; Durand et al., 1996). The idea that an increase in the number of silent synapses could provide a structural platform for the incorporation of additional AMPA receptors and hence a boost in LTP expression is corroborated by similar observations in CamKIV^−/−^ mice acutely expressing constitutively active CamKIV variants (Marie et al., 2005) and observations in PSD95^−/−^ mice (Huang et al. 2015). A selective decrease in the number, but not the strength, of mature (AMPA receptor-containing) synapses is in good agreement with the results of a recent study on the effects of lentiviral-mediated SHANK2 knockdown in hippocampal slice culture (Shi et al., 2017) and relates to the reduction in frequency, but not amplitude, of spontaneously occurring mEPSCs in Shank2 Δex7^−/−^ mice (Schmeisser et al., 2012).

Why synaptic transmission is weak in Shank2 Δex7^−/−^ mice, although LTP can readily be induced *in vitro* and *in vivo*, remains to be elucidated. Similar observations have been made, however, upon acute knockdown of PSD-95 in hippocampal slice cultures (Ehrlich et al., 2007). It is conceivable that the loss of SHANK2 may be a reason for decreased synaptic stability (Stanika et al., 2015) or the failure to instruct concomitant structural changes in potentiated synapses along with the early insertion of AMPA receptors (MacGillavry et al., 2016). Another open question is why, in our hands, AMPA/NMDA ratios in wild-type versus knockout animals are dependent on slice storage conditions. It is tempting to speculate that the hyperplasticity of synapses in Shank2 Δex7^−/−^ mice could influence their recovery after slice preparation.

At first sight, it seems peculiar that the Shank2 Δex7^−/−^ mouse line stands alone with its phenotype of increased LTP, while mouse lines with mutations in other Shank homologs show either no change in synaptic plasticity (Shank1, Hung et al., 2008) or reduced LTP (Shank3, Bozdagi et al., 2010; Kouser et al., 2013). However, considering the vast body of literature on isoform-specific expression (Lim et al., 1999); protein–protein interactions (Lim et al., 2001; Boeckers et al., 2005); and spatiotemporal localization of SHANK1, SHANK2, and SHANK3 (Böckers et al., 2004; Grabrucker et al., 2011), different phenotypes in mutants lacking different isoforms are not surprising (Shi et al., 2017) and may in fact be indicators of isoform-specific functions of SHANK proteins in synapse formation, development, and plasticity.

Of note, Shank2 Δex6-7^−/−^ mice (Won et al., 2012) do not show increased *in vivo* LTP in our hands, in contrast to Shank2 Δex7^−/−^ mice. This difference between the two models is in line with earlier reports on their excitatory synaptic transmission (Schmeisser et al., 2012; Won et al., 2012) and parallels differences in their GABAergic physiology (Lim et al., 2017). When comparing these two Shank2 knockout mouse lines, isoform-specific differences in protein function fall short of explaining phenotypic differences, since both knockouts are null mutants on the SHANK protein level (Schmeisser et al., 2012; Won et al., 2012). The two mouse models exhibit differences in their genetic background, however, manifest in the differential expression of numerous genes (Lim et al., 2017). Resultant genetic interactions could be partly responsible for phenotypic variations and are in line with the supposed existence of “modifier genes” in the pathophysiology and etiology of ASDs (Leblond et al., 2012).

Shank2 Δex7^−/−^ mice reproduce several phenotypes associated with ASDs, a neurodevelopmental disorder (Schmeisser et al., 2012; Won et al., 2012; Ko et al., 2016). In this context, it is interesting that we have uncovered an unexpected neurodevelopmental phenotype in Shank2 Δex7^−/−^ mice: defective synapse maturation. A similar excess of silent synapses has been described in mice lacking Sapap3 (Wan et al., 2011), a GKAP family protein that directly interacts with SHANKs (Boeckers et al., 1999; Naisbitt et al., 1999; Yao et al., 1999). Of note, the loss of Sapap3 causes obsessive-compulsive behavioral traits in mice (Welch et al., 2007), which are typical in individuals with autism and have also been described in Shank2 Δex7^−/−^ mice (Schmeisser et al., 2012). Likewise, FMR1^−/−^ mice, a model for the autism-related fragile X syndrome, show altered plasticity and synapse maturation in the barrel cortex (Harlow et al., 2010). Last, in a mouse line with SYNGAP1 haploinsufficiency, a genetic model for intellectual disability and ASDs, hippocampal synapses are unsilenced prematurely, adversely impacting learning and memory in the adult animal (Rumbaugh et al., 2006; Clement et al., 2012). Together with the present findings, these studies draw a picture of synapse maturation as a tightly controlled process, the dysregulation of which seems of relevance for a range of neurodevelopmental disorders; the process of synaptic maturation should therefore be investigated further in future studies, in particular with regard to how it can be influenced by therapeutic approaches.

## Synthesis

Reviewing Editor: Christian Hansel, University of Chicago Decisions are customarily a result of the Reviewing Editor and the peer reviewers coming together and discussing their recommendations until a consensus is reached. When revisions are invited, a fact-based synthesis statement explaining their decision and outlining what is needed to prepare a revision will be listed below.

Wegener et al. provide experiments demonstrating that defects in long- term plasticity are observed in Shank2e7KO mice in vivo. In addition, they provide a mechanistic explanation for this phenotype. Mutations associated with SHANK are associated with ASD in humans and lead to complex phenotypic outcome in mouse models. Uncovering the role associated with specific Shank mutation in vivo is therefore essential to further our understanding of the pathological condition. In their manuscript the authors describe experiments in which they analyze LTP in the hippocampus of Shank2-/- mice. There is some contradictory data in the published literature from prior studies by this group which found enhanced LTP and the findings of Won et al 2012 which demonstrated a deficit in hippocampal LTP. In an attempt to address these inconsistencies the authors preform further LTP experiments in vitro using methods more in line with Won et al, and also perform in vivo LTP experiments in the mice. They find that LTP is enhanced in these experiments and basal transmission is reduced. While consistent with their prior work this does not get to the question of why there are contradictory data in another mouse line. Therefore this finding does little to move the field forward or explain and unify the findings in Shank2 mutants.

Major:

The authors present minimal synaptic stimulation experiments, which demonstrate an abundance of silent synapses in young Shank2-/- mice. The authors claim that the increased abundance of silent synapses is a “plausible mechanistic explanation” for the increased LTP. However a major concern is that the silent synapse experiments were performed in 3-4 week old mice and the LTP experiments in 8-9 week old mice. This undercuts the authors' contention that these two correlated findings have a causal link. The abundance of silent synapses changes over the course of development and it is possible that in 8-9 week old mice there will be very few/none in both genotypes. The authors must perform both LTP experiments in young mice (3-4 weeks) when there is a big difference in silent synapse number, and also perform minimal stimulation experiments at 8-9 weeks when the enhanced LTP phenotype is apparent.

Wegener et al. overstate an apparent contradiction between two Shank2 knockout mice (e6-7 KO and e7 KO. I have some concerns regarding the comparison that is made with other Shank2 models as there are key information from the literature that need further consideration.

There are many potential explanations for the discrepancy of results between the Won et al. and the Schmeisser et al. results. One of them is that although very similar, the genetic strategies used in the two studies are not the same. I think this could be better explained in the current manuscript. This topic has been comprehensively reviewed in Jiang and Ehlers in 2013, a study of this kind could be referenced. For the same reason of clarity it is important to mention in the introduction that a direct comparison between the Shank2 knockout mice (e6-7 KO and e7 KO) has indeed be conducted (Lim et al. 2017) showing that there are indeed measurable physiological differences between the two lines. These information pieces are important for the conclusion that the authors make regarding the comparison of the different models.

Together with LTP, LTD is also a discrepancy between the two articles. Wegener et al. decided to focus solely on LTP for their in vitro analysis, is there any reasons for it? I would suggest to determine if the difference in protocol could alter LTD in wild type and Shank2-/- mice. In their conclusion, the author also forget to take in account the fact that GABA receptor-mediated inhibitory neurotransmission observed in Shank2e6-7KO and not in Shank2e7 KO. One could imagine that a defect of GABAergic maturation in one model and not the other could partly explain the difference in excitatory synaptic transmission.

In Figure 1C, the authors state in the legend that they re-analyzed Schmeisser et al data at P25. The authors should explain that in the results sections as well as the motivation to do it. This is a discrepancy with the original Schmeisser et al publication but also the Won et al article that needs to be discussed. In line 184 of the manuscript the authors state that the reduction of the AMPA/NMDA ratio is reduced in certain conditions. What are these conditions? Is it simply the difference between the two Shank2KO models or are the authors referring to experimental conditions?

In the discussion the authors “warrant caution in generalizing strategies like targeting NMDA receptors or boosting LTP” and referred to Won et al 2012 and Bozdagi et al 2013. These authors never claimed that such strategies could be generalized but stated that “mGluR5 may be a novel target for the treatment of ASD involving altered NMDAR function” (Won et al 2012) and that “One interesting outcome of the current study is that compounds that enhance glutamatergic transmission, including those that specifically enhance AMPA transmission (AMPAkines) could possibly represent therapeutic approaches in these conditions” Bozdagi et al 2013. Wegener et al should moderate their comment or at least not cite these authors improperly.

Minor points:

In Figure 3B the authors should show the NMDA/AMPA ratio and not the AMPA/NMDA, simply for consistency with the presentation in the Schmeisser et al article.

The authors refer to their models as Shank2-/- but I think that according to the current state in the literature it would be preferable to refer it to as Shank2e7KO.
